# Adolescent sleep and its disruption in depression and anxiety

**DOI:** 10.3389/fnins.2024.1479420

**Published:** 2024-11-07

**Authors:** Ruiming Chai, Wen-Jie Bian

**Affiliations:** ^1^Westlake Laboratory of Life Sciences and Biomedicine, Hangzhou, China; ^2^School of Life Sciences, Westlake University, Hangzhou, China; ^3^Institute of Biology, Westlake Institute for Advanced Study, Hangzhou, China

**Keywords:** adolescent sleep, sleep disruption, depression, anxiety, neural development

## Abstract

Adolescence is a pivotal stage during development when one’s personality, emotion, and behavioral traits are shaped to a great extent, and the underlying neural circuits undergo substantial developmental organizations. Dramatic and dynamic changes occur in sleep architecture throughout the postnatal developmental course. Insufficient sleep and disruption of sleep/wake coherence are prevalent among the adolescents worldwide, and even so in young patients with neuropsychiatric conditions. Although accumulating evidence has suggested a tight association between sleep disruption and depression/anxiety, the causal relationship remains largely unclear. More importantly, most of these studies focused on adult subjects, and little is known about the role of sleep during the development of mood and behavior. Here we review recent studies investigating the acute and chronic effects of adolescent sleep disruption on depression and anxiety both in humans and rodent models with focuses on the assessment methodology and age. By discussing the findings and unsolved problems, we hope to achieve a better understanding of the relationship between sleep and mental health in adolescents and provide insights for future research.

## Introduction

Adolescence is the transition period between childhood and adulthood when individuals experience significant physiological and psychological transformations. It is believed to be a critical period for higher-order brain functions such as learning, decision-making, emotion, and behavior. It is also a “risk” stage for many neuropsychiatric disorders, including major depressive disorders, anxiety disorders, alcohol and other substance abuse, schizophrenia, and bipolar disorder (Davidson et al., 2015; Solmi et al., 2022). The neural underpinnings of this susceptibility remain largely unknown. It likely involves the delayed maturation, and thus prolonged vulnerability, of brain structures underlying these higher-order functions, such as the prefrontal cortex (PFC) (Kolk and Rakic, 2022). Following the rapid formation of massive synaptic connections in childhood, the adolescent brain undergoes substantial refinement and modification of neural connections, therefore exhibits a high level of plasticity and hence more vulnerable to environmental changes compared to the adult brain (Kolk and Rakic, 2022; Moyer and Zuo, 2018). This was mostly demonstrated in the sensory-motor cortices, but evidence suggesting other cortical regions, such as the PFC, as well as subcortical regions follow the similar developmental trajectory, although the time course may be different (Kolk and Rakic, 2022).

Sleep architecture evolves dramatically during postnatal development (Roffwarg et al., 1966). The total amount of sleep decreases progressively and dramatically from 14-16 h/day in newborn babies to 7–8 h/day in adults. Almost 50% of total sleep at birth (~ 8 h/day) is the rapid-eye-movement (REM) sleep, which is rapidly reduced in childhood, and by the onset of adolescence, it constitutes of 20% of total sleep (~2 h/day). On the other hand, non-REM (NREM) sleep first increases in infancy and remains at relatively high level (~9 h/day), before it demonstrates a substantial decrease during adolescence and eventually contributes to 80% of adult sleep (~6 h/day). In addition to the sleep duration changes, specific brain activity patterns also demonstrate dynamics over the adolescent developmental course. Compared to early adolescence, the EEG delta power in NREM sleep and theta power in REM sleep decrease by about 60% at the end of adolescence (Campbell and Feinberg, 2009). These changes occur about 1 year earlier in girls than in boys, likely due to the advanced puberty onset in females (Campbell et al., 2012). Sleep spindles also show decrease in amplitude but increase in frequency, density, and coherence during adolescence (Herrera and Tarokh, 2024). Recent surveys suggest that although sleep pattern varies cross-nationally, insufficient sleep is prevalent among adolescents world-wide especially on the school days, with the percentage of adolescents meeting sleep recommendations (8 h/day) (Hirshkowitz et al., 2015) ranging from 32 to 86% (Gariepy et al., 2020; Zhang et al., 2023). Transformations of lifestyle and physiology greatly shape sleep schedule during adolescence, and adolescents start to show a rapidly increasing risk of sleep problems. A natural shift in circadian rhythm when entering puberty leads to delayed bed time by ~2 h, while the sleep need is not reduced (Crowley et al., 2007). This may cause “delayed sleep phase syndrome” which is more common among adolescents than in adults (Gariepy et al., 2020; Wheaton and Claussen, 2021). As teenagers grow up, increasing social activities lead to “social jet lag” and irregular sleep schedule (Hasler et al., 2022). The relationship between sleep and mental health has long been postulated based on the high occurrence of sleep problems in psychiatric disorders (Ford and Kamerow, 1989). Recent findings further underscore a developmental link given the prevalence of sleep disturbances in neurodevelopmental disorders, such as autism and schizophrenia, and in neuropsychiatric disorders not typically considered a developmental disorder but having a high risk of disease onset during adolescence, such as depression and anxiety disorders; in many cases, sleep disturbances precede the progression of psychiatric illness and are thought to be a cause of mood dysregulation and behavioral abnormalities (Hertenstein et al., 2019).

## Sleep disruptions in depression and anxiety in humans

Sleep disturbances have long been viewed as one of the core symptoms of depression (Ford and Kamerow, 1989; Jindal and Thase, 2004). In fact, doctors may not be confident to diagnose depression in the absence of sleep complaints. Common sleep disturbances linked to depression include insomnia (80%), hypersomnia (15%), and obstructive sleep apnea (20%) (Zhang et al., 2022). About 40% of insomnia patients are also clinically diagnosed with depression, suggesting a bidirectional association (Hertenstein et al., 2019; Zhang et al., 2022). Interestingly, patients with depression might oscillate between insomnia and hypersomnia within the same depressive episode, highlighting the complexity of this relationship. Like depression, patients with anxiety disorders frequently experience insomnia, including increased subjective sleep disturbance, reduced sleep continuity, and decreased total sleep time (Alfano et al., 2009).

In a study that sampled 87 adolescents (age 12–17) and 88 younger children (age 6–11) (Alfano et al., 2009), depressive symptoms were more strongly associated with sleep problems in adolescents, whereas anxiety appeared to be linked to sleep issues across all youth. The authors generated a single sleep problems factor that contained sleep-related items extracted from the Child Behavior Checklist (CBCL), Revised Child Anxiety and Depression Scales (RCADS) and Revised Children’s Manifest Anxiety Scale (RCMAS) and also consistent with other commonly used measures of childhood sleep (e.g., Children’s Sleep Habits Questionnaire), including overtiredness, less (or more) sleep than most children, trouble sleeping, and worries about going to bed at night. They found in both age groups, the sleep problems factor had a strong correlation with the anxiety measures, including generalized anxiety, panic and agoraphobia, and social anxiety in RCADS. However, for depression measures using the Child Depression Inventory (CDI), sleep problems showed a higher correlation coefficient in the adolescent group than in the children group. A large-sample survey in junior high/high school teenagers in Japan presented interesting sex differences in sleep habits and the associations with depression/anxiety that female adolescents generally sleep less than male adolescents (by ~20 min) and that their sleep duration with the lowest risk of depression/anxiety is also less (by 50–60 min) (Ojio et al., 2016). In addition, more insomnia, hypersomnia, or nightmares, shorter REM sleep latency and increased rapid eye movement activity were reported in suicidal psychiatric patients than the nonsuicidal ones, and suicide is perhaps the most devastating consequence of depression that significantly increases during adolescence (Liu and Buysse, 2006). Furthermore, longitudinal studies found that the association between sleep problems and depression/anxiety increased in adolescence as compared to that in early childhood and that a set of sleep variables at age 15, including total sleep time on school days, daytime sleepiness, and night waking, predicted anxiety and depression symptoms prospectively at later stages, either by clinic assessment or self-report questionnaires (Orchard et al., 2020; Gregory and O'Connor, 2002). Roberts and Duong showed that increased risk of major depression by reduced sleep may in turn increase the risk of further sleep decrease in a one-year follow-up study, indicating a reciprocal relationship (Roberts and Duong, 2014). In contrast, Wang et al. found that sleep problems in adolescence (age 14–17) can be retrospectively predicted by anxiety/depression symptoms in childhood (age 5), but not vice versa (Wang et al., 2016). These correlation studies demonstrate tight yet intricate associations between sleep disturbances and depression/anxiety in a developmental context (for a systematic review, see Zhang et al., 2022).

In experimental settings, acute or chronic sleep deprivation (SD) has been shown to cause immediate mood disruption, psychological distress and depressive- and anxiety-like symptoms both in adults (Talbot et al., 2010; Dinges et al., 1997; Babson et al., 2010) and in adolescents (Talbot et al., 2010; Dagys et al., 2012; Baum et al., 2014). In contrast, interestingly, SD was initially suggested as an alleviating method for depression by Johann Christian August Heinroth in 1818, who was considered the first university professor of psychiatry (Steinberg and Hegerl, 2014). This idea, named “SD therapy,” was supported by clinical reports and experimental trials subsequently at some level (Tse et al., 2024; Ioannou et al., 2021; Giedke and Schwarzler, 2002; Hemmeter et al., 2010). One-time total SD or repeated sleep restriction over days (a couple of hours per day) seemed effective either as add-ons to standard treatments for depression or as single-component therapy (Tse et al., 2024; Ioannou et al., 2021; Giedke and Schwarzler, 2002; Hemmeter et al., 2010). This discrepancy may be due to a difference in the subjects: the SD therapy was used in patients already diagnosed with depression or insomnia, or both, whereas SD with mood-disrupting consequences was mostly done in healthy subjects. However, the mechanisms underlying these opposite mood effects remain largely unknown and warrant more investigation. Recent mechanistic research in humans and mouse models added another layer of mystery to this paradoxical role of sleep in depression/anxiety (please see *Sleep deprivation to alleviate depression/anxiety* in the next section).

## Sleep disruptions and depression- and anxiety-like behaviors in rodents

A role of sleep in regulating mood has emerged from studies in rodent models over the past decades. However, before we discuss the findings, special consideration must be given to the disruption methods, manipulation ages and potential confounding factors.

### Disruption methods and duration

Up until now, multiple sleep disruption or deprivation methods have been developed. Perhaps one of the simplest methods is to expose animals to novel objects/environments or running wheels and utilize their voluntary exploration or exercise to keep them awake. This method is known to cause minimal stress (Kopp et al., 2006). However, mice can quickly become habituated to the stimulus, and thus the deprivation usually is no longer effective after the initial 1–2 h. Another manual deprivation method is by “gently touching” the animal when it displays signs of sleep. However, this protocol requires continuous monitoring of the mouse behavior and/or EEG, and the amount of “touches” required to keep wakefulness can increase substantially after the initial 1–2 h. Thus, this technique is labor-intensive and difficult to keep consistent across experimenters, making it challenging for chronic SD. Automated SD, on the other hand, has been widely adopted in long-term experiments. The setups typically involve forced motion or alertness to prolong wakefulness from hours to days, by using devices such as moving treadmills or rotating wheels (Colavito et al., 2013), small alternating platforms (Pierard et al., 2007), spinning disks (Rechtschaffen et al., 1983), or grid floors above water (Shinomiya et al., 2003). Recent studies used programmed shaking platform (Bian et al., 2022; Lord et al., 2022; Bian and De Lecea, 2023) or moving bars (Puech et al., 2023) showed effective deprivation for 4 h or more. Furthermore, ways to selectively deprive a sleep state (i.e., NREM or REM) are still limited. REM-specific deprivation (REM-D) has been reported, but the efficacy and specificity are not satisfactory. For example, REM-D using the platform-over-water method (Morden et al., 1967) in mice only resulted in ~65% reduction of REM sleep and a significant reduction of NREM sleep (Arthaud et al., 2015). NREM-specific disruption, on the other hand, is more challenging, as NREM reduction will normally be followed by REM depletion. NREM fragmentation without affecting REM sleep, however, has been achieved by optogenetic methods (Rolls et al., 2011).

### Experimental age

In mice, adolescence roughly corresponds to between postnatal day (P) 23 and P60 (Brust et al., 2015). Sleep studies in developing mice has been facing the challenges of polysomnographic recording in young pups, due to their small and fragile skulls as well as the limited recovery time allowed between the surgery and desired recording age. Therefore, the amount of sleep deprived were often measured using behavioral criteria instead, which are less accurate and unable to identify sleep states or specific components. The rapid growth of developing skull also makes longitudinal recording difficult. Recently, researchers have managed to record EEG/EMG in post-weaning mice (Medina et al., 2022). However, aside from the tolerance issue, the isolation required by polysomnographic recording with cable transmission may cause potential problems, as juvenile social isolation has been found to cause significant changes in sociability (Yamamuro et al., 2020) and sleep patterns (Sotelo et al., 2024). Wireless polysomnography allowing sleep recording in the litter will potentially provide a solution to this dilemma, although further optimization toward lighter headset and longer recording time is needed. EEG/EMG in neonatal rats are much easier. Indeed, a lot of insights regarding sleep’s role in early development came from rat studies (Frank, 2020). Nonetheless, the isolation problem remains, and the lack of genetic tools has limited mechanistic interrogations in rats.

### Stress as a confounding factor

SD has long been recognized to induce stress, which is signified by the elevated stress hormones in the blood (e.g., cortisol in humans, corticosterone in rodents) and can dramatically alter emotion, behavior, or sleep itself (Nollet et al., 2020). The stress brought about by SD comes from two major sources. One is the physical stimulation applied to keep the animal awake in the experimental apparatus, e.g., platform shaking or bar rotating, and can be controlled through proper experimental design. The other source is the loss of sleep *per se*. Prolonged sleep loss causes significant corticosterone elevation in rats and eventually leads to pre-mature death even when the physical stimulation was controlled (Rechtschaffen et al., 1983), suggesting that sleep alone plays an essential role in maintaining physiological integrity. In the context of development, if sleep disruption contributes to depression and anxiety, is this effect merely a consequence of compromised physiology, or mediated by distinct mechanisms? To answer this question, it is vital to dissect the contribution of sleep in a setting without systematic stress response, which is challenging and often omitted by previous studies. It should be noted that SD is not necessarily accompanied by a detectable stress response. Whether a SD protocol is stressful or not largely depends on the disruption method and duration as well as the manipulation age (Nollet et al., 2020). SD methods like exposing to novel objects (Kopp et al., 2006) and gentle handling (Vecsey et al., 2013; Hagewoud et al., 2010) caused no increase in plasma corticosterone level, or only a mild increase to which the animal can quickly habituate, and therefore are not considered significant stressors. Carefully designed setups using running wheels, shaking/rotating platforms, or closed-loop disruptions can also achieve sleep loss without inducing significant corticosterone increase (Bian et al., 2022; Lord et al., 2022; Bian and De Lecea, 2023; Nollet et al., 2020). In addition, setting up proper stress control [e.g., restrain stress (Bian et al., 2022) or direct injection of corticosterone (Yang et al., 2014)] and manipulating individual sleep components without changing the overall sleep amount also help to exclude the confounding effects from the behavioral results (Bian et al., 2022). Furthermore, the long-lasting feature of depression and anxiety as a mood suggests that the underlying neurobiological changes should be persistent. Therefore, after SD manipulation is removed but before behavioral assessment, allowing sleep to recover (or not) may be a good strategy to exclude the immediate stress influence and examine the long-term effects of SD. Notably, the magnitude of corticosterone increase induced by SD is more severe in neonatal rats than in adolescents despite a developmental increase in baseline corticosterone level, suggesting developmental endurance of SD stress (Hairston et al., 2001).

### Depression- and anxiety-like behavior caused by adolescent SD

To assess anxiety-like behavior, open field (OFT), elevated plus maze (EPM), light/dark chamber (LD) tests are commonly used (Crawley, 2007). These tests compare the time that mice spent in the “low-danger” area (edges in OFT, closed arms in EPM, or dark chambers in LD) over the “high-danger” area (center area in OFT, open arms in EPM, or light chambers in LD) as an index of anxiety level. To assay different aspects of depression, multiple behavioral tests have been developed. Increased immobility time in the forced swimming test (FST) is considered a sign of despair in rodents, whereas decreased preference for sucrose over water in the sucrose preference drinking test (SPT) reflects anhedonia (Crawley, 2007). In addition, sucrose negative contrast test (SNCT) measures the response change to reward downshift, and the depressed animals may exhibit hypo- or hypersensitivity to the shifting stimuli (Matthews et al., 1996). Self-grooming in the sucrose splash test (SST) is considered to reflect self-care motivation, the impairment of which indicates depressive state (Lefter et al., 2020).

As summarized in Table 1, behavioral changes reflecting a depressive and anxious mood have been reported following SD at different developmental stages. Early adolescent SD in rats using rotating bars (6–8 h/day, P19–P32) were reported to increase anxiety, as measured by EPM and LD, and decrease motivation for self-care and increase despair as measured by SST and FST, respectively (Atrooz et al., 2019; Atrooz et al., 2022). These behavioral changes were likely long-lasting as some of them were observed at later timepoints approximately 1 or 2 months after SD. However, in the study the corticosterone level was not examined, therefore the confounding stress cannot be excluded. In another rat study, 18 h/day REM-D for a longer period in adolescence (P21–P42) caused similar reduction in open-arm time and entries in EPM at P49, without changing the reward response downshift in SNCT (da Silva et al., 2018), but the corticosterone level was also increased by REM-D. Deprivation of either total or REM sleep at an earlier childhood stage (P14–21) in mice, rats or prairie voles returned negative results on anxiety-like behavior (Lord et al., 2022; Feng and Ma, 2003; Jones et al., 2020), with the mouse and vole studies showing no significant stress, suggesting the anxiety-inducing effect may be specific to adolescent SD. In mice, long-term, mild SD from early neonatal stage all the way to mid/late-adolescence (3 h/day by gentle handling, P5–P42/52) did not cause an increase in corticosterone level, but decreased locomotion, which might be considered a sign of depression, although the anxiety level in EPM seemed unchanged (Sare et al., 2016; Sare et al., 2019). Depressive-like behaviors, on the other hand, were induced by only 8 days of SD of similar intensity by non-stressful gentle-handling method in mice during P42–49, including longer immobility time and shortened immobility latency in FST, although stress was not excluded in this study (Murack et al., 2021). These behavioral changes did not occur when the same SD protocol was performed in adult animals, indicating a development-specific impact (Murack et al., 2021). Notably, when rats were subjected to early adolescent SD (P19–32), more anxiety-like behavior were displayed immediately after SD (P33) and at young adult stage (P60), but not at the later stage (P90), whereas depression-like behavior was more dramatic at P90, but not at earlier stages (Atrooz et al., 2019; Atrooz et al., 2022). This temporal dissociation of anxiety- and depression-like states indicate in-parallel mechanisms caused by adolescent SD or differential susceptibilities of the symptoms. Alternatively, there may be a mechanistic link between the two sets of behavioral abnormalities that sustained anxiety, caused by SD, leads to depression.

**Table 1:** Effects of developmental SD on anxiety and depression in rodent studies.

References	Animal model	Deprivation method	Disrupted sleep component	Manipulation age, duration per day	Observation age	Anxiety-like	Depression-like	Other behavior impact	Serum corticosterone measurement
Sare et al. (2016, 2019)	Mouse	Gentle handling	Nonspecific	P5 - P42 /P52 3 h/day	P43–45, P72–76 /P42-52, P84-94	OFT: no change in center/total ratio EPM: no effect	OFT: activity↓ (P44, P73/P42, 84)	Sex-specific effects in social behavior	Not in the 2016 study, but in the 2019 study, no significant change
Lord et al. (2022)	Mouse (wild-type and Shank3 ΔC)	Orbital shaker	Nonspecific	P14–P21 (P56–63 as adult control) Full-day	P70–P115	EPM: Time in open arms different between genotypes but not changed by SD	OFT: activity different between genotypes but not changed by SD		Yes, no significant change
Bian et al. (2022)	Mouse	Programmed shaker	Nonspecific	P35–42	P56	Locomotor activity: no effect EPM: no effect		Social novelty preference↓	Yes, no significant change
Murack et al. (2021)	Mouse	Gentle handling	Nonspecific	6–7 weeks (10–11 week as adult control) 4 h/day	7 weeks	Not tested	FST: immobility time↑, latency↓(7 week)		Measurements were done after restraint. SD mice show greater stress response in females. Stress by SD was not measured
Feng and Ma (2003)	Rat	Shaker controlled by REM detection	REM	P14–P21, full-day	3 months	Not tested	OFT: no effect	Sexual behavior↓ Shock-induced fighting: offensive behavior↑ and defensive behavior↓ REM in adulthood↑	No
Atrooz et al. (2019, 2022)	Rat	Rotating bar	Nonspecific	P19–P32, 6–8 h/day	P33, P60, P90	EPM: time and entries in open arms↓ (P33, P60) LD: time in lit area ↓(P33)	FST: immobility time↑ (P90) SST: time grooming↓(P33)	Alcohol preference↑ (P39) indicating increased reward-seeking behavior	No
da Silva et al. (2018)	Rat	Platforms immersed in water	REM	P21–P42 18 h/day	P45–49	EPM: time and entries in open arms↓ (P49)	SNCT: no effect on SCI (P45–48)		Yes, higher in SD group
Jones et al. (2019, 2020)	Prairie vole	Orbital shaker	Nonspecific	P14–P21 full-day	P80–P130	LD: no effect	No effect on baseline ethanol intake Ethanol intake following foot shock↑	Interaction with novel objects↓ Social bonding↓	Yes, no significant change

### Adolescent SD-induced social deficits and their relationship with depression and anxiety

Social withdraw and difficulties in social communication are a core symptom shared by many neuropsychiatric disorders, including depression, anxiety disorders, autism and schizophrenia. Interestingly in several studies above mentioned, there was a striking social phenotype. Feng and Ma showed that REM-D in pre-weaning male rats (P14–21) decreased their mounting latency when facing female conspecifics in adulthood, but intromission and ejaculation were not affected (Feng and Ma, 2003). However, maternal separation alone produced similar deficits in the same study, making it less clear how much sleep loss contributes to this defect. These REM-deprived rats also displayed decreased aggression in shock-induced fighting (Feng and Ma, 2003). In voles, early-life SD led to decreased social bonding (Jones et al., 2019). Saré et al. performed daily gentle handling SD (3 h/day) in mice during P5–42 and found that it led to slight but significant increase in sociability immediately after SD and in social novelty preference 4 weeks later (Sare et al., 2016; Sare et al., 2019). However, when SD was prolonged to P52 (Sare et al., 2019), or performed only during middle (Bian et al., 2022) or late adolescence (Lord et al., 2022), it seemed only to impair social novelty preference. Notably, this effect is not likely due to stress or juvenile social isolation as these confounding factors were carefully avoided or controlled in one of these studies (Bian et al., 2022). Interestingly, although loss of social interest is often viewed as a sign of depression in human patients, adolescent SD-induced lack of social novelty preference was not likely due to depression, based on the following observations (Bian et al., 2022). Firstly, the overall sociability was not changed in SD mice. Secondly, the level of rewarding dopaminergic activity, including the activation of dopaminergic neurons in the ventral tegmental area (VTA) and the dopamine release in the nucleus accumbens (NAc), was not reduced; it was the timing of these dopaminergic activities in relation to social interaction events that was impaired in SD mice. Thirdly, SD mice exhibited normal craving for food and sucrose, and their VTA response to these non-social rewards was not significantly changed, suggesting the absence of general anhedonia. Finally, EPM results suggest SD mice had normal anxiety level compared to the control group. Therefore, the impact of adolescent sleep on mood and behavior is multi-layered, and detailed dissection of intricate effects and mechanisms of SD is warranted.

### Sleep deprivation to alleviate depression/anxiety

In contrast to the perspective that long-term sleep disruption leads to heightened risk of depression and anxiety, transient SD has been shown clinically a rapid and efficient way to alleviate depression with a success rate of 60–70%, better than any existing anti-depressant drugs (Tse et al., 2024; Boland et al., 2017). SD increases the homeostatic sleep drive and thus, supposedly leads to a more consolidated recovery sleep afterwards. However, this does not likely play a role here because the mood response occurs, if it does, within hours after SD treatment begins, and in fact, more than 80% of the responders show a relapse into depression after the recovery sleep (Hemmeter et al., 2010). Human fMRI revealed that one night of total SD enhanced connectivity between the amygdala and anterior cingulate cortex (ACC), which is associated with a better mood and impaired in depressed patients, mimicking anti-depressant effects (Chai et al., 2023). Research in mice showed that 12 h SD was sufficient to significantly reduce the immobility time in FST, as well as that in a tail suspension test (TST), another assay for depression, and that activation of astrocyte adenosine signaling mediated this SD effect (Hines et al., 2013). Enhanced level of mood-boosting neuromodulators, such as dopamine and serotonin, has been suggested to account for SD’s anti-depressant effect (Wu et al., 2024; Bjorvatn et al., 2002), but more complex changes are likely at the receptor level (Tuan et al., 2023; Vaseghi et al., 2023), making it still far from conclusive whether SD promotes or impairs dopaminergic and serotonergic neurotransmission. Interestingly, 6 h SD by gentle handling was shown to inhibit neuroinflammation and improving neuroplasticity in the ACC (Shi et al., 2023) and 12 h SD by a rotating bar enhanced dopamine signaling in the mesocorticolimbic but not nigrostriatal pathway (Wu et al., 2024) (but corticosterone level was not examined in this study), whereas a stressful 72-h SD protocol resulted in systematic dopaminergic maladaptation and enhanced neuroinflammation in the brain (Tuan et al., 2023). These findings offer mechanistic insights into the different impacts of acute versus chronic SD in the brain, especially in the structures heavily implicated in depression/anxiety, such as the limbic system. Yet the evidence so far is still limited, and many questions remain unanswered. How does the acute changes in the affected brain regions lead to more systematic deficits when SD is prolonged? How does the brain process the accumulating sleep pressure (and the stress that comes with it) and the mood boost? Can SD truly enhance mood, or is it merely a rapid state transition with no lasting benefits or even detrimental outcomes later? These unresolved questions necessitate further investigation, and the answers may in turn help understanding the long-term effects of SD in adolescence.

## Conclusion and future directions

The prevalence of sleep disruptions among adolescents, particularly those with neuropsychiatric conditions, is a global concern. While we have begun to understand the acute and chronic effects of sleep disruptions on mood, emotion, and behavior, much remains unknown about the long-term impact of these disruptions on the developing brain, both under physiological and pathological conditions. Key questions remain unanswered regarding to what extent sleep disruption contributes to a specific symptom in a particular disorder, whether sleep disruption is more “disruptive” during development than in adulthood, whether the impact is long-lasting and/or reversible, and what sleep components, brain regions and cell types play major roles. Future research should aim to address these gaps in our understanding. SD experiments in adult rodents have revealed global changes in multiple neural or non-neural pathways that may serve as the underlying mechanisms. However, due to challenges in implementing circuit-specific measurement and manipulation methods in developing animals, our understanding of the intricate role of sleep in development is still in its early stages. Our recent work utilizing viral tracing, fiber photometry and optogenetic tools demonstrates that SD during a critical, mid-adolescent period impairs social behavior by overexciting the developing VTA dopaminergic circuits and disrupting their developmental wiring (Bian et al., 2022). We further identified NREM slow waves to be a major contributing sleep component (Bian et al., 2022). Recent development of non-invasive, ultrasound stimulation techniques further offers great potentials for circuit- or neuronal type-specific interrogations during adolescence (Murphy et al., 2024; Murphy et al., 2022). There is also a need for more comprehensive and systematic understanding based on the dissection of contributions of sleep components (SWA, spindles, etc.) and neural circuits in depression and anxiety, as well as novel tools to facilitate this work. The answers will help achieve a better understanding of the relationship between adolescent sleep and mental health, and ultimately, develop more effective intervention methods for adolescents suffering from sleep disruptions and neuropsychiatric disorders.
